# Revision of the United States Pharmacopœia

**Published:** 1860-03

**Authors:** Geo. B. Wood

**Affiliations:** President; Philadelphia


REVISION OF THE UNITED STATES PHARMACOPŒIA.—It will be seen from the following circular, signed by Dr. George B. Wood, that the Convention for revising our National Pharmacopœia, will be held at Washington City on Wednesday, the 2d of May next.

The following appointments of delegates to the Convention for revising the Pharmacopœia, to meet at Washington on the first Wednesday in May next, having been duly made known to me, are hereby announced in compliance with a provision of the convention of 1850.

From the Massachusetts College of Pharmacy, Messrs. Theodore Metcalf and Charles T. Carney; from the New York Academy of Medicine, B. W. McCready, M.D., E. H. Davis, M.D., and E. R. Squibb, M.D.; from the College of Physicians of Philadelphia, Geo. B. Wood, M.D., R. P. Thomas, M.D., and Robert Bridges, M.D.; from the University of Pennsylvania, Jos. Carson, M.D., R. E. Rogers, M.D., and Jos. Leidy, M.D.; from the Jefferson Medical College of Philadelphia, Franklin Bache, M.D., and T. D. Mitchell, M.D.; from the Philadelphia College of Pharmacy, Messrs. Wm. Procter, Jr., Edward Parrish, and Alfred B. Taylor; from the Medical Society of the State of North Carolina, Wm. George Thomas, M.D., Peter E. Hines, M.D., and Edward Warren, M.D.; from the Medical Society of the State of New York, Drs. E. R. Squibb, Howard Townsend, and Caleb Green; from the College of Pharmacy of the City of New York, Messrs. Wm. Hegeman, Alex. Cushman, and John Meakim; from the Faculty of Physic of the University of Maryland, Professors Samuel Chew, Charles Frick, and Wm. E. A. Aikin; from the Maryland College of Pharmacy, Messrs. G. M. Andrews, Israel J. Graham, and Alpheus P. Sharp; and from the Cincinnati College of Pharmacy, Messrs. E. S. Wayne, W. S. Merrell, and W. J. M. Gordon.

By order of the Convention of 1850.
GEO. B. WOOD, *President.*

PHILADELPHIA, February 14, 1860.

[Medical journals will please copy.]

